# EPN (*O*-ethyl-*O*-(4-nitrophenyl)­phenylthiophosphonate): MAK Value Documentation, addendum – Translation of the German version from 2024

**DOI:** 10.34865/mb210464e11_2ad

**Published:** 2026-06-30

**Authors:** Andrea Hartwig, MAK Commission

**Affiliations:** 1 Institute of Applied Biosciences, Department of Food Chemistry and Toxicology, Karlsruhe Institute of Technology (KIT) Adenauerring 20a, Building 50.41 76131 Karlsruhe Germany; 2 Permanent Senate Commission for the Investigation of Health Hazards of Chemical Compounds in the Work Area, Deutsche Forschungsgemeinschaft, Kennedyallee 40, 53175 Bonn, Germany. Further information: Permanent Senate Commission for the Investigation of Health Hazards of Chemical Compounds in the Work Area | DFG

**Keywords:** EPN (O-ethyl-O-(4-nitrophenyl)­phenylthiophosphonate), insecticide, pesticide, toxicity, evaluation, acetylcholinesterase inhibitor

## Abstract

EPN (*O*-ethyl-*O*-(4-nitrophenyl)phenylthiophosphonate) [2104-64-5] is used as an in­secticide and acaricide but is not approved in the European Union. The previous MAK value documentation and addendum do not reflect the current data situation of the substance. The MAK Commissiondecided that a new evaluation is not of high priority. The MAK value and the other classifications are therefore suspended and the substance is listed in the Section II c of the List of MAK and BAT Values for substances no longer evaluated.

**Table:** 

**MAK value**	**see Section II c of the List of MAK and BAT Values**
**Peak limitation**	**–**
	
**Absorption through the skin**	**–**
**Sensitization**	**–**
**Carcinogenicity**	**–**
**Prenatal toxicity**	**–**
**Germ cell mutagenicity**	**–**
	
**BLW (2023)**	**reduction of the acetylcholinesterase activity in erythrocytes to 70% of the reference value^a)^**
	
Synonyms	*O*-ethyl *O*-(4-nitrophenyl) phenylphosphonothionate
Chemical name (IUPAC)	ethoxy-(4-nitrophenoxy)-phenyl-sulfanylidene-λ^5^-phosphane
CAS number	2104-64-5
Molar mass	323.30 g/mol
Melting point	36 °C (NCBI 2023)
Vapour pressure at 25 °C	1.27 × 10^–6^ hPa (NCBI 2023)
log K_OW_	4.78 (NCBI 2023)
Solubility	3.11 mg/l water (NCBI 2023)

^a)^ The BLW (biological guidance value) is derived as the ceiling value because of acute toxic effects.

This addendum was prepared because the previous evaluations no longer reflect the data currently available for the MAK value and for the designations and classifications of the substance. EPN (*O*-ethyl-*O*-(4-nitrophenyl)phenylthiophosphonate) is an insecticide and acaricide from the class of organophosphates (AERU 2022). The substance is a cholinesterase inhibitor that is activated by metabolism in the liver. The biological guidance value (BLW) for acetylcholinesterase inhibitors (reduction of the acetylcholinesterase activity in erythrocytes to 70% of the reference value; Lewalter 1995; Weistenhöfer et al. 2024) therefore applies to EPN. The BLW is derived as the ceiling value because of acute toxic effects. However, it was not investigated whether this is the most sensitive end point.

In 1958, a MAK value of 0.5 mg/m³ I (inhalable fraction) was set and EPN was designated with an “H” (for substances which can be absorbed through the skin in toxicologically relevant amounts). In 2002, the substance was assigned to Peak Limitation Category II with an excursion factor of 2 (Greim 2002, available in German only; Henschler 1972, available in German only).

EPN has never been approved for use in the European Union (European Commission 2022; European Parliament and European Council 2009). EPN is not included in the report on the approval history and regulations for plant protection products published by the Federal Office of Consumer Protection and Food Safety in 2010. It can therefore be assumed that the active substance has also never been approved for use in the Federal Republic of Germany and was not used in the former German Democratic Republic (BVL 2010).

The previous evaluations (MAK value documentation and addendum) do not reflect the currently available data. However, a re-evaluation of the substance is not a priority. Therefore, the MAK value, the peak limitation and the “H” designation have been withdrawn and EPN has been allocated to Section II c of the List of MAK and BAT Values (DFG 2022). This section lists substances for which the previous MAK values, designations and classifications have been withdrawn and which are no longer being reviewed at present.
